# All-angle acoustic topological insulators: from omnidirectional antennas to angle-tunable Klein tunneling

**DOI:** 10.1093/nsr/nwag346

**Published:** 2026-06-05

**Authors:** Zichong Yue, Shuochen Wang, Zhiwang Zhang, Danwei Liao, Haixiao Zhang, Ying Cheng, Xiaojun Liu, Johan Christensen

**Affiliations:** Department of Physics, MOE Key Laboratory of Modern Acoustics, Collaborative Innovation Center of Advanced Microstructures, Nanjing University, Nanjing 210093, China; Department of Physics, MOE Key Laboratory of Modern Acoustics, Collaborative Innovation Center of Advanced Microstructures, Nanjing University, Nanjing 210093, China; Department of Physics, MOE Key Laboratory of Modern Acoustics, Collaborative Innovation Center of Advanced Microstructures, Nanjing University, Nanjing 210093, China; Department of Physics, MOE Key Laboratory of Modern Acoustics, Collaborative Innovation Center of Advanced Microstructures, Nanjing University, Nanjing 210093, China; Department of Physics, MOE Key Laboratory of Modern Acoustics, Collaborative Innovation Center of Advanced Microstructures, Nanjing University, Nanjing 210093, China; School of Electrical and Information Engineering, Changzhou Institute of Technology, Changzhou 213032, China; Department of Physics, MOE Key Laboratory of Modern Acoustics, Collaborative Innovation Center of Advanced Microstructures, Nanjing University, Nanjing 210093, China; Department of Physics, MOE Key Laboratory of Modern Acoustics, Collaborative Innovation Center of Advanced Microstructures, Nanjing University, Nanjing 210093, China; IMDEA Materials Institute, Madrid 28906, Spain

**Keywords:** acoustic metamaterials, topological acoustics, Dirac cone, omnidirectional acoustic antenna

## Abstract

Conventional Dirac cones are tightly pinned at high-symmetry points in reciprocal space, although they can be slightly shifted through straining or stretching. In this work, we discuss an approach to translate Dirac cones across the entire Brillouin zone of a 2D topological sonic metamaterial that is based upon a tilted Su–Schrieffer–Heeger model. By judiciously engineering the associated hopping amplitudes, one can readily alter the positions of the Dirac cones, thereby broadening the possibilities for a wide range of applications and phenomena. For example, we present an experimental demonstration of out-coupled nontrivial edge states, capable of radiating in any direction. Additionally, we use this approach to demonstrate Klein tunneling effects that can be achieved at arbitrary angles of incidence. We anticipate that our findings will expand the prospect of both fundamental and applied topological physics.

## Teaser

This study provides an all-angle acoustic topological insulator supporting tunable Dirac cones in reciprocal space, enabling sound radiation in all directions and Klein tunneling effect at arbitrary angles.

## INTRODUCTION

Dirac cones (DCs) are distinctive features in the electronic band structures of materials such as graphene and topological insulators and they give rise to unique electron transport properties, including the quantum Hall effect [1], quantum spin-Hall effect [2], quantum valley-Hall effect [3], topological insulators [4], and Zitterbewegung [5] etc. Leveraging mesoscopic scales, precise fabrication and measurement technologies, topological metamaterials serve as a superior platform to mimic Dirac-like conical dispersions and explore otherwise hard to access complex physical phenomena [6–12]. For example, the phenomenon of acoustic Klein tunneling was observed experimentally based on frequency-shifted Dirac band structures [11]. Topological insulators are closely related to the Dirac dispersion where breaking the time-reversal or other spatial symmetries lifts its degeneracy, which enables topological phase transitions that have been thoroughly discussed in acoustic settings comprising multidimensional topological boundary states in both Hermitian and non-Hermitian systems [13–29].

Conventional DCs are confined to high-symmetric points of the first Brillouin zone (BZ), such as the K and K$^\prime$ points in a honeycomb lattice [30,31]. E.g., the degeneracy of DCs in phononic lattices can be lifted by breaking the mirror symmetry through tunable mass modulation [32] or employing a reconfigurable negative capacitance circuit [33], comprising topological valley-Hall edge states. Further, non-local interactions beyond nearest neighbor coupling lead to the formation of additional DCs in the BZ [34–36]. Recent studies have shown that introducing uniaxial deformation in strained honeycomb lattice can create a synthetic gauge field [37], causing slight variations in the positions of DCs, resulting in the emergence of discrete Landau levels and associated edge states [38–44]. However, it is important to highlight that this adjustable feature only allows for limited tunability, which significantly limits their versatile applications for wave steering and manipulation. Consequently, achieving traversal DCs across the entire BZ using classical waves, where their positions can be freely adjusted, remains a formidable challenge.

**Figure 1.**
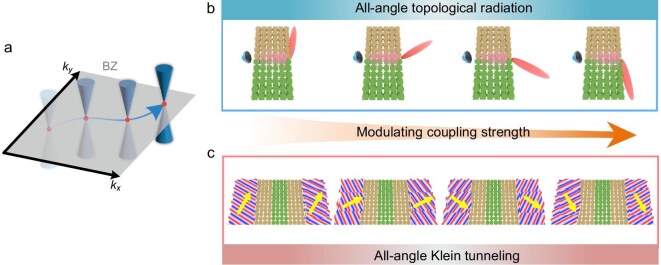
Traversable DC across the entire BZ. (a) Schematic of the paired DCs whose positions can be changed by modulating the geometrical parameters. (b) Topological antenna with tunable emission angle, and (c) Klein tunneling from any direction.

In this study, we present a 2D topological sonic metamaterial that is based on the titled 2D Su–Schrieffer–Heeger (SSH) model that accommodates a pair of DCs within the first BZ. By adjusting the geometrical parameters and coupling strengths, the DCs can be flexibly translated, exhibiting characteristics akin to a topological semimetal (Fig. 1a). Using this approach, we experimentally report a directional acoustic antenna with tunable radiation angles based on a valley-Hall topological insulator (Fig. 1b). The emitted beam can be engineered to radiate in any direction ranging from $-\rm {90}^{\circ }$ to $\rm {90}^{\circ }$, offering distinct advantages beyond angles fixed at the K/K$^\prime$ valleys [18]. Furthermore, we also demonstrate that translated DCs facilitate all-angle Klein tunneling of which the working angle of incidence can be modulated at will (Fig. 1c).

## RESULTS

### Movable DCs in 2D tilted acoustic SSH model

**Figure 2.**
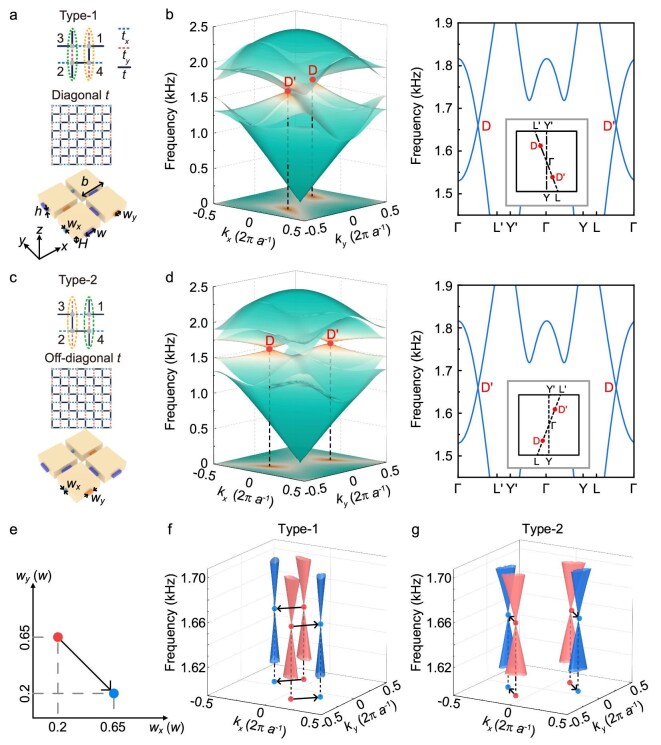
Displaced DCs in a 2D tilted acoustic SSH model. (a) Interpretations of the tight-binding models (top two panels) and corresponding SC (bottom panel) for the 2D tilted SSH model consisting of the diagonal coupling pattern (type-1). (b) Calculated acoustic band diagram supporting a pair of DCs. (c and d) Same as (a and b), but inverting the coupling pattern to the off-diagonal one to form the type-2 tilted SSH model. (e) The route along which the coupling strength is changed. (f and g) With the parameter variations in (e), the positions of the DCs are shifted accordingly for the type-1 (f) and type-2 (g) SCs.

We begin with the 2D SSH model, a renowned topological model hosting multiple topological boundary states [45–47]. The integration of diagonally and off-diagonally distributed coupling patterns are introduced as illustrated in Fig. 2a and c, respectively, representing the geometric interpretation of the tight-binding model (TBM) [48]. For simplicity, we denote these two lattices as type-1 and type-2, and we emphasize that the distinction between type-1 and type-2 lattices lies in the spatial orientation of the constant couplings *t*. To be specific, the couplings *t* extend from bottom-left to top-right directions in the type-1 lattice, which are complemented by two variable couplings $t_x$ and $t_y$ alternatingly distributed, as illustrated in Fig. 2a. In contrast, the constant couplings *t* in the type-2 lattice are arranged in an off-diagonal pattern extending from top-left to bottom-right orientation instead (see Fig. 2c). The effective Hamiltonian of the type-1 lattice can be expressed as


(1) \begin{eqnarray*}
H(\mathbf {k}) &=&
\left(\begin{array}{cc} 0 &\quad q(\mathbf {k})\\
q^{\dagger }(\mathbf {k}) &\quad 0 \end{array}\right),\\
{{\rm with} } \quad q(\mathbf {k}) &=&
\left(\begin{array}{cc}
q_{13} &\quad q_{14} \\
q_{23} &\quad q_{24} \end{array}\right)\\
&=&
\left(\begin{array}{cc}
t_{x}+t e^{i k_{x}} &\quad t+t_{y} e^{i k_{y}}\\
t_{y}+t e^{-i k_{y}} &\quad t+t_{x} e^{-i k_{x}} \end{array}\right),\\
\end{eqnarray*}

where $q_{13}$, $q_{14}$, $q_{23}$, and $q_{24}$ represent the terms interacting between different lattice sites 1–3, 1–4, 2–3, and 2–4, respectively. Comparing the geometric interpretations shown in Fig. 2a and c, we find that the couplings between sites 2–3 and between sites 1–4 are inverted in these two situations. In the type-1 lattice, the intra-cell and inter-cell couplings between sites 2 and 3 are $t_y$ and *t*, respectively, which, however, are changed to be *t* and $t_y$ in the type-2 lattice. A similar transformation also occurs between sites 1 and 4 as labeled by dashed frames with different colors in Fig. 2a and c. Consequently, we demonstrate that the difference between the type-1 (diagonal) and type-2 (off-diagonal) lattices is reflected only in the terms $q_{23}$ and $q_{14}$ of the corresponding Hamiltonian, while the other two terms $q_{13}$ and $q_{24}$ remain the same. The Hamiltonian of the type-2 lattice can accordingly be derived as


(2) \begin{eqnarray*}
q(\mathbf {k}) =
\left(\begin{array}{cc}
t_{x}+t e^{i k_{x}} &\quad t_{y}+t e^{i k_{y}} \\
t+t_{y} e^{-i k_{y}} &\quad t+t_{x} e^{-i k_{x}} \end{array}\right).
\end{eqnarray*}

Further details on the TBM and its link with the acoustic model can be found in Section S1.

To build this tilted 2D SSH model in acoustics, we design the corresponding type-1 and type-2 sonic crystals (SCs) consisting of the tube-coupled sound cavities as illustrated in the bottom panels of Fig. 2a and c, respectively. For type-1 SC, each unit cell contains four identical cuboid sound cavities, with a lattice constant of $a=76.8$ mm. The side length and the height of each cuboid are $b=32$ mm and $H=12$ mm, respectively. The coupling tubes, rectangular in cross-section with a uniform height $h=3.5$ mm, offer modulatable coupling strengths through variations in widths *w*, $w_x$, and $w_y$. We demonstrate that the paired DCs not only appear in this system, but also can be movable and traverse the entire BZ through tuning the coupling strengths. For instance, setting the parameters as $w=12$ mm, $w_x=0.2w$, and $w_y=0.65w$, a pair of DCs are found at D $(-0.08,0 .23)\frac{2\pi }{a}$ and D$^\prime (0.08,-0.23)\frac{2\pi }{a}$ in the reciprocal space, as can be seen from the calculated band diagram in Fig. 2b. Interestingly, once the diagonally distributed coupling patterns (type-1) are inverted to the off-diagonal ones (type-2) as depicted in Fig. 2c with other geometric parameters unchanged, the positions of the DCs are shifted to D $(-0.08,-0.23)\frac{2\pi }{a}$ and D$^\prime (0.08,0.23)\frac{2\pi }{a}$, which are symmetric with the previous ones along the $k_y$ axis (Fig. 2d).

Employing these SC types, the DCs can be moved to arbitrary positions in the *k* space (see Section S2). To shed more light on these mobile properties, we outline the coupling strength’s evolution along the track, illustrated in Fig. 2e, where red and blue dots mark the starting [$(w_x,w_y) = (0.2w,0.65w)$] and terminal [$(w_x,w_y) = (0.65w,0.2w)$] points, respectively. The trajectories of the DCs for type-1 and type-2 SCs are clearly illustrated in Fig. 2f and g respectively, which plot the simulated band diagrams around the Dirac frequency. Simulation details are expounded in Section S3. It should be noted that there are various practical approaches to dynamically adjusting the coupling strength, as elucidated in Section S4. Leveraging acoustic first-order resonance, real-time fine-tuning of coupling strength becomes feasible by adapting the heights of movable coupling tubes. Thanks to these adaptable DCs, versatile acoustic functionalities can be achieved. In the following, we realize a single‐beam full‐space scanning topological acoustic antenna, from which the radiated direction of sound waves can be modulated toward any desired angle.

### Topological single‐beam acoustic antenna

**Figure 3.**
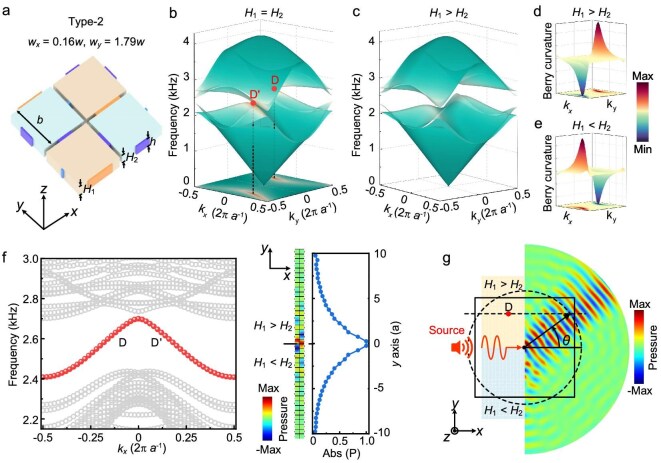
Topological directional acoustic antenna based on the tilted 2D SSH model. (a) Schematic of the unit cell and (b) band diagram for the type-2 tilted 2D SSH SC with $w_x = 0.16w$ and $w_y = 1.79w$ while keeping $H_1=H_2$. (c) Band diagram and (d) calculated Berry curvature below the band gap for the structure with $H_1 =$ 13.2 mm and $H_2 =$ 10.8 mm. (e) Berry curvature for the structure with $H_1 =$ 10.8 mm and $H_2 =$ 13.2 mm instead. (f) Simulated dispersion relations of the ribbon-shaped SCs with a *x*-direction interface between two kinds of SCs. Right panel indicates the acoustic eigenmode profile of the topological edge states at $k_x = -0.16 \frac{2\pi }{a}$. (g) Simulated distributions of the radiated pressure fields at the frequency of $f = 2.6$ kHz. The *k*-space analysis on the out-coupling of the valley-projected edge states from the interface is also illustrated.

Considering the sample fabrications and experimental measurements, we optimize the geometric parameters of the structure to $h =$ 10.8 mm and $b =$ 36 mm instead. Figure 3a describes the unit cell of the type-2 SC with $w_x =0.16w$ and $w_y =1.79w$. The calculated band diagram in Fig. 3b indicates a pair of DCs located at D $(-0.17,0.34)\frac{2\pi }{a}$ and D$^\prime$  $(0.17,-0.34)\frac{2\pi }{a}$, which verifies the validity of the system under the geometric optimization. Breaking the inversion symmetry is achieved by setting different heights for the cavities in the unit cell. Specifically, two orange-colored cavities possess the heights of $H_1=13.2$ mm, and the other two turquois cavities are set as $H_2=10.8$ mm as illustrated in Fig. 3a. This gives rise to the degeneracy breakdown of the DCs, resulting in the formation of the valley states alongside a complete band gap as seen in Fig. 3c. Furthermore, we calculate the Berry curvature through the following efficient method [49]:


(3) \begin{eqnarray*}
\tilde{F}_{12}(k_x,k_y) &\equiv& \ln U_1(k_x,k_y)U_2(k_x + \Delta k_x,k_y)\\
&&\quad U_1^{-1}(k_x,k_y + \Delta k_y)U_2^{-1}(k_x ,k_y) ,\\
U_1(k_x,k_y) &=& \langle n(k_x,k_y) \vert n(k_x + \Delta k_x,k_y ) \rangle ,\\
U_2(k_x,k_y) &=& \langle n(k_x,k_y) \vert n(k_x ,k_y + \Delta k_y ) \rangle ,
\end{eqnarray*}

where $\tilde{F}_{12}(k_x,k_y)$ represents the Berry connection with $n(k_x,k_y)$ for the wave eigenfunctions extracted from the eigenfrequencies calculations of unit cell. From the derived results in Fig. 3d, we find two sharp peaks located around two DCs but with opposite amplitudes, which indicates the zero Chern number due to the preservation of the time-reversal symmetry. However, if we focus on one single DC only and integrate the Berry curvature in half of the first BZ, the non-zero valley-Chern numbers can be obtained as ${C}^ {\rm D}_v = 1/2 $ and $C^{{\rm D}^\prime }_v = -1/2 $, leading to the topological nontrivial valley-Hall phases. When modulating the heights of the cavities to $H_1 < H_2$ with $H_1 = 10.8 $ mm and $H_2 = 13.2$ mm instead, the shape of the band diagram remains unchanged (not shown here), but the peak values of the Berry curvature are inverted as shown in Fig. 3e, which results in the valley-Chern numbers of $C^{\rm D}_v = -1/2 $ and $C^{{\rm D}^\prime }_v=1/2 $. According to the bulk-boundary correspondence, topological edge states are expected to exist along the interface separating the two topologically distinct regions.

Figure 3f shows the dispersion relations for a ribbon-shaped SC, where the Floquet boundary conditions are applied on the left and right sides. A topological interface separates 10 units with $H_1 > H_2$ from another 10 units with $H_1 < H_2$. The contrast in valley-Chern numbers, $\Delta C^{\rm D}_v = 1$, gives rise to forward-propagating edge states around valley D, as evidenced by the pressure distributions of the eigenstates in the right panel of Fig. 3f. It has been proven that the topological edge states projected by the valleys at the high-symmetric points of BZ facilitate out-coupling from the topological interface into the free space [18]. Here, we examine the out-coupling effect of the topological edge states originating from the unpinned valleys. As depicted in Fig. 3g, a finite 2D SC is built based on the aforementioned structures, and sound waves are launched from left to right to excite the D valley-projected topological edge states. Equifrequency curves in free space (black dashed circle) and the first BZ (black solid square) are plotted to showcase the radiation angle. Quantitatively, the theoretical radiation angle $\theta _{\rm {theory}}$ can be determined through the wave vector matching mechanism ${\bf k}\cdot {\bf e}_{\rm {term}} = {\bf D}\cdot {\bf e}_{\rm {term}}$, resulting in $\theta _{\rm {theory}} = \frac{180}{\pi }\arcsin (\frac{k_yc}{af}) = 36.8^{\circ }$ at the frequency $f = 2.6$ kHz. The simulated radiative pressure fields in Fig. 3g reveal a highly directional sound beam, with the resulting radiation angle of $\theta _{\rm {sim}} = 36.8^{\circ }$ closely matching the theoretical predictions. As a result, we exemplify that the DCs unpinned at high-symmetric points could be utilized to achieve directional sound beam out-coupling. Subsequently, we will provide guidelines for designing a full-space scanning topological acoustic antenna with customizable radiation angles.

### Full-space beam scanning via tunable DCs

**Figure 4.**
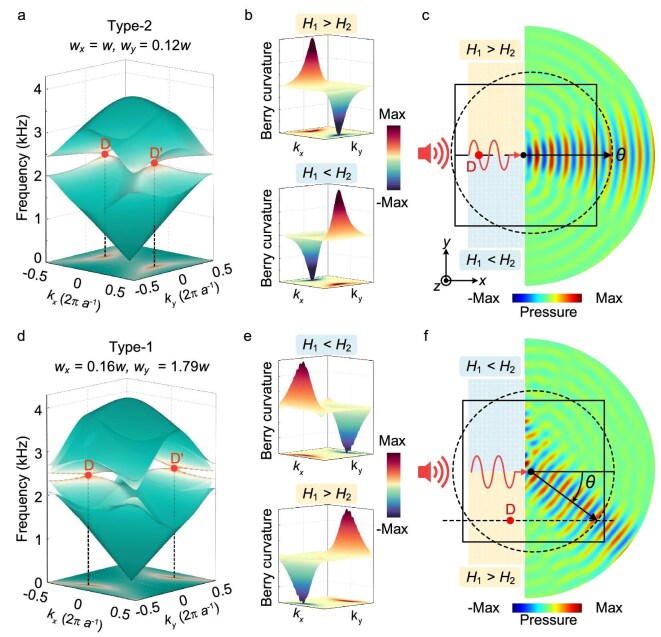
Tuning the radiative directivity at will through displaced DCs. (a) Band diagram for the type-2 SC with $w_x =w$, $w_y =0.12w$ and $H_1=H_2$, which supports the DCs located at D $(-0.28,0)\frac{2\pi }{a}$ and D$^\prime$  $(0.28,0)\frac{2\pi }{a}$. (b) Calculated Berry curvature for the inversion symmetry-broken structures with $H_1 > H_2$ (top panel) and $H_1 < H_2$ (bottom panel). (c) Simulated distributions of the radiated pressure fields at the frequency of $f = 2.6$ kHz using the topological SCs discussed in (b). (d–f) Same as (a–c), but for the type-1 SC with $w_x = 0.16w$ and $w_y =1.79w$ instead, which supports the DCs located at D $(-0.17,-0.34)\frac{2\pi }{a}$ and D$^\prime$  $(0.17,0.34)\frac{2\pi }{a}$.

We demonstrate that the radiation angle of the antenna can be modulated by moving the DCs within the BZ. Two specific scenarios are presented to demonstrate the near-zero and negative radiation angles. For a type-2 tilted SC with $w_x =$  *w* and $w_y =$ 0.12*w*, a pair of DCs are located at D $(-0.28,0)\frac{2\pi }{a}$ and D$^\prime$  $(0.28,0)\frac{2\pi }{a}$ as displayed in Fig. 4a. The band degeneracies are then broken by introducing differences in cavity heights $H_1$ and $H_2$. The calculated Berry curvatures below the band gap for structures with $H_1 > H_2$ (top panel) and $H_1 < H_2$ (bottom panel) are shown in Fig. 4b. The opposite peak values guarantee the existence of the topological valley-Hall edge states. Additional details on the dispersion relations of the ribbon-shaped structures can be found in Section S5. Since the Dirac cone D lies on the $k_x$ axis, the theoretical radiation angle can be determined as $\theta _{\rm {theory}} = 0^{\circ }$ at the frequency of $f=2.6$ kHz, as validated by simulations in Fig. 4c. Employing a similar approach, directional radiation with a negative angle of $\theta _{\rm {theory}} = -36.8^{\circ }$ can be achieved by relocating the DCs to points D $(-0.17,-0.34)\frac{2\pi }{a}$ and D$^\prime$  $(0.17,0.34)\frac{2\pi }{a}$. This transition involves adjusting the couplings to a type-1 pattern and fine-tuning the geometric parameters to $w_x = 0.16w$ and $w_y = 1.79w$, as detailed in Fig. 4d–f.

### Experimental demonstration and robustness analysis

**Figure 5.**
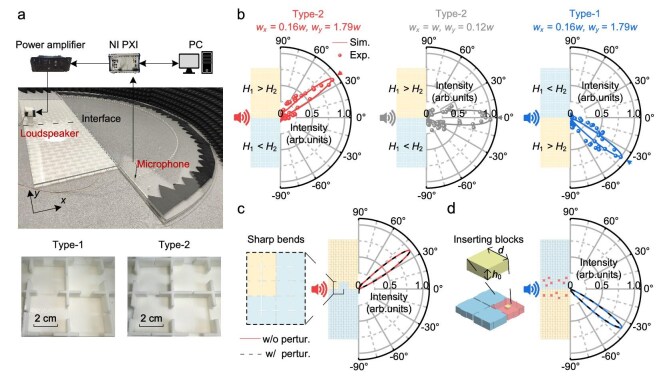
Experimental demonstrations and robustness analysis. (a) Photograph of the experimental set up. Bottom panels show the enlarged views of the fabricated SC units. (b) Simulated (curves) and experimentally measured (dots) far-field radiation patterns of the topological acoustic antenna working at different directional angles. The arrows label the theoretical predictions. (c and d) Robustness of the functional device considering the randomly distributed perturbations with (c) sharp bends and (d) inserting extra blocks into cavities. The solid and dashed curves represent the simulated results with and without perturbations, respectively.

To experimentally validate the tunable radiation angles induced by the traversal DCs, we construct an experimental setup as shown in Fig. 5a, which includes a finite $6a\times 20a$ topological SC and a semicircular 2D free space. The far-field radiation patterns of the acoustic intensity fields are detected by scanning microphone placements at $2^\circ$ intervals. Detailed experimental procedures are outlined in Section S3. Figure 5b presents the simulated and experimentally measured results for three scenarios discussed in Fig. 3g, 4c, and 4f. The sound radiation directionality is presented in the polar coordinate, and the curve represents the normalized intensity of $p^2(\theta )/{\rm max}[p^2(\theta )]$ with $p(\theta )$ for the detected sound pressure at different angular coordinates while scanning the far-field patterns. The experimental data exhibit strong agreements with simulations and theoretical predictions, with noticeable suppression of side lobes resulting in a collimated single-beam radiation pattern. Analysis of the influence of inherent loss can be found in Section S6. Thanks to the flexible tunability of the DCs’ positions, we emphasize that any desired radiation angle can be achieved. Moreover, we further explore cases with larger radiation angles of up to $\pm 90^\circ$ in Section S7 and the details of simulations can be found in Section S3. As the hallmark and defining feature of topological insulators, the proposed device exhibits remarkable resilience against potential perturbations that may arise during fabrication and routine operations. Figures 5c and d depict two key perturbations within the device, namely sharp bends and randomly distributed disordered cavities created by inserting acoustically rigid cubic blocks sized at $d=8$ mm and $h_0=3.5$ mm. Examination of the nearly unchanged far-field patterns (dashed curves) compared to pristine cases (solid curves) reveals the robustness of radiation intensity and directionality against these disturbances. More discussions about the robustness analysis can be found in Section S8.

### Incidence-sensitive acoustic Klein tunneling effect

**Figure 6.**
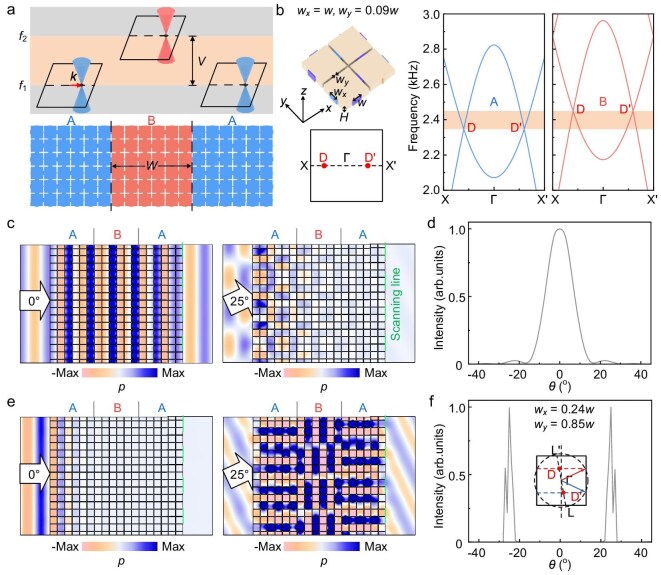
Directional sound Klein tunneling. (a) Schematic diagram of the sound Klein tunneling effect. (b) Unit cell of SC (left panel) and the calculated band diagrams for the different SCs (right panel). The shaded area indicates the potential barrier. (c) Simulated acoustic absolute pressure fields for 0° and 25° incidence at $f=2390$ Hz. (d) Intensity transmission along the dashed line in (c) as a function of the incident angle. (e and f) Same as (c and d) but for the structures with different parameters at frequency of $f=2478$ Hz, which lead to the moved DCs and the Klein tunneling effect under other incident angles. Arrows in the inset of (f) represent the incident angle directions which could excite the acoustic Klein tunneling effect.

Klein tunneling effect has been experimentally verified in acoustics supporting nearly perfect transmissions in the range of potential barrier [11]. Inciting this phenomenon requires the incident wave direction with a wave vector parallel to the direction along the central point towards the DCs in the BZ. Nevertheless, in previously documented cases, DCs have been predominantly fixed at the high-symmetric points of the first BZ, such as the K and K$^\prime$ points in the honeycomb lattice. Consequently, the incident angle for Klein tunneling remains unadjustable, significantly limiting its practical applications. We demonstrate that the above limitations could be resolved by using our designed SCs which host the movable DCs in the entire BZs, enabling direction-tunable sound Klein tunneling. As shown in Fig. 6a, a 2D acoustic heterostructure is constructed by two types of titled SCs labeled as A and B, each with distinct Dirac frequencies $f_1$ and $f_2$, respectively. Both SC types share identical parameters of $w_x = w$ and $w_y = 0.09w$, yet differ in heights: $H =$ 14 mm for SC-A and $H =$ 12 mm for SC-B. The width of the potential barrier is $W=3a$. In this case, the paired DCs are located along the $\Gamma$–X direction at D $(-0.3,0)\frac{2\pi }{a}$ and D$^\prime$  $(0.3,0)\frac{2\pi }{a}$ as shown in Fig. 6b, resulting in a potential barrier of $V=f_2-f_1=2447$ Hz $- 2347$ Hz $=100$ Hz. To assess the incident sensitivity of acoustic Klein tunneling, pressure field distributions for $0^\circ$ and $25^\circ$ incident angles are visualized in Fig. 6c. It is obvious that most of the energy transmits through the acoustic heterostructure at a $0^\circ$ incident angle, while being forbidden at a $25^\circ$ incident angle. Averaged transmitted intensity along with varying incident angles is detected via a green dashed line, with the corresponding outcomes shown in Fig. 6d, revealing a distinct peak at $\rm {0}^{\circ }$ aligning well with expectations. Furthermore, we modify the geometric parameters to $w_x = 0.24w$ and $w_y = 0.85w$, leading to the DCs appearing at D $(-0.04,0.26)\frac{2\pi }{a}$ and D$^\prime$  $(0.04,-0.26)\frac{2\pi }{a}$ as shown in Fig. 6e and f. More simulations considering the inherent loss can be found in Section S6. Notably, at $0^\circ$ incidence, acoustic wave transmission is suppressed through the heterostructure, contrasting with near-perfect transmission at a $25^\circ$ incident angle (Fig. 6e). To shed light on the coupling efficiency during Klein tunneling, we discuss the associated wave-vector matching condition as illustrated with the equal-frequency contour in Fig. 6f. Further insights into the transmission behavior at different incident angles are depicted in Fig. 6f, affirming the applicability of the direction-tunable sound Klein tunneling effect. In Section S9, the transmission coefficients vs. incident angles for the sound Klein tunneling effect are also carefully studied.

## DISCUSSION

In conclusion, we have shown an approach that lets the DCs traverse the entire BZ in a tilted 2D SSH superlattice formed by interconnected cavities. By adjusting the coupling patterns and strengths, the DCs can be translated across the entire BZ. Leveraging this method, we design a topological directional acoustic antenna with adjustable radiation angles, whose performance is validated through experimental measurements of far-field radiative patterns. Our device maintains negligible side lobes even at large angles and exhibits strong robustness against perturbations. Further, we also numerically discuss Klein tunneling and how it maintains high performance with sound coming from any direction. We stress that the ability to translate DCs across the entire BZ offers promising opportunities beyond the two applications discussed in our work. In fact, the movable DCs enable the realization of various topological acoustic phenomena that are no longer constrained to specific high-symmetry points. This flexibility paves the way for designing topological devices with functionality that is robust against the direction of incidence or propagation. For instance, key components such as topological valley filters, valves, and diverers, which traditionally rely on fixed valleys in momentum space [50], can now be reconfigured to operate efficiently at arbitrarily chosen incident angles or propagation directions. Moreover, this approach may facilitate the development of omnidirectional topological waveguides, angle-tunable beam splitters, and reconfigurable acoustic logic elements, all of which benefit from the momentum-space tunability enabled by our platform. An example of wave vector-tunable valley filter is further studied in Section S10. Importantly, our methodology is transferable to a variety of physical systems, offering opportunities for the exploration of intriguing functionalities through movable DCs.

## Supplementary Material

nwag346_Supplemental_File
