# Supplementary material for: All-angle acoustic topological insulators: from omnidirectional antennas to angle-tunable Klein tunneling
Source: Natl Sci Rev. 2026 Jun 5;13(15):nwag346. doi: 10.1093/nsr/nwag346 (PMC13455640; doi:10.1093/nsr/nwag346)
Supplement: Supplementary file 1 [file nwag346_supplemental_file.pdf]

Supplementary Material for:
All-angle Acoustic Topological Insulators: from Omnidirectional Antennas to Angle-tunable Klein Tunneling

Zichong Yue,[1] Shuochen Wang,[1] Zhiwang Zhang,[1,*] Danwei Liao,[1]
Haixiao Zhang,[1,2] Ying Cheng,[1,†] Xiaojun Liu,[1,‡] and Johan Christensen[3,§]

[1]*Department of Physics, MOE Key Laboratory of Modern Acoustics,*
Collaborative Innovation Center of Advanced Microstructures,
Jiangsu Physical Science Research Center,
Nanjing University, Nanjing 210093, China

[2]*School of Electrical and Information Engineering,*
Changzhou Institute of Technology, Changzhou 213032, China

[3]*IMDEA Materials Institute, Calle Eric Kandel, 2, 28906 Getafe, Madrid, Spain*

[*] zhangzhiwang@nju.edu.cn
[†] chengying@nju.edu.cn
[‡] liuxiaojun@nju.edu.cn
[§] johan.christensen@imdea.org

Sect. 1. TIGHT-BINDING APPROACH AND ITS LINK WITH THE ACOUSTIC MODEL OF TILTED 2D SSH LATTICE

The tight-binding models of the proposed tilted SSH lattices are illustrated in Fig. S1, of which the effective Bloch Hamiltonian reads

$$H(\mathbf{k}) = \begin{pmatrix} 0 & q(\mathbf{k}) \\ q^{\dagger}(\mathbf{k}) & 0 \end{pmatrix}. \tag{1}$$

For the type-1 tilted 2D SSH model as shown in Fig. S1a, the hopping with a constant strength of $t = 1$ spreads continuously from the bottom left to the top right, which satisfies

$$q(\mathbf{k}) = \begin{pmatrix} t_x + te^{ik_x} & t + t_y e^{ik_y} \\ t_y + te^{-ik_y} & t + t_x e^{-ik_x} \end{pmatrix}. \tag{2}$$

Conversely, for the case shown in Fig. S1c, the constant hopping extends from the top left to the bottom right instead, known as the type-2 tilted 2D SSH model with

$$q(\mathbf{k}) = \begin{pmatrix} t_x + te^{ik_x} & t_y + te^{ik_y} \\ t + t_y e^{-ik_y} & t + t_x e^{-ik_x} \end{pmatrix}. \tag{3}$$

Here, $\mathbf{k} = (k_x, k_y)$ is the wave vector, while t_x and t_y denote the adjustable hopping amplitudes along the x and y directions. The calculated band diagram for the type-1 tilted SSH model with t_x = 0.2 and t_y = 0.65 is shown in Fig. S1b. Notably, a pair of DCs emerge in the BZ, and these DCs are not pinned to high-symmetric points. For the type-2 tilted SSH model, the band diagram in Fig. S1d reveals the presence of a pair of DCs as well. However, the locations of DCs differ from those of the type-1 model, exhibiting symmetry along the coordinate axis. Subsequently, the coupling strengths are modulated from values indicated by orange dots (t_x = 0.2 and t_y = 0.65) to those marked by green dots (t_x = 0.65 and t_y = 0.2) along the illustrated route in Fig. S1e. The corresponding paths showing the variations in the DCs' locations within the BZ are shown in Fig. S1f, where the solid dots and hollow circles represent the type-1 and type-2 lattice, respectively. Specifically, the band diagrams for the four structural configurations while scanning along the dotted BZ path (as depicted in Fig. S1f) are presented in Fig. S1g, revealing four distinct DCs situated at varying positions.

Moreover, we solve the eigenvalue problem based on our proposed acoustic model through an equivalent transmission line approach. We demonstrate that interconnecting coupling waveguides

can be equivalent as acoustic masses of $L_i = \rho_0 l_i/S_i$ with $i = t, t_x$ and t_y for different couplings, where $S = h \times w$ represents the cross-section area of the tubes. l_i, h and w indicate the length, height and width of the tube. The sound cavities can be treated as equivalent acoustic capacitance of $C_0 = V/\rho_0 c_0^2$ with V, ρ_0 and c_0 for the volume of cavity, mass density and velocity of air. Then we apply Kirchhoff's current law at the first cavity of the unit cell as

$$\frac{i}{\omega L_t}\left(p_{\mathbf{r}}^4 - p_{\mathbf{r}}^1\right) + \frac{i}{\omega L_t}\left(p_{\mathbf{r+x}}^3 - p_{\mathbf{r}}^1\right) + \frac{i}{\omega L_{tx}}\left(p_{\mathbf{r}}^3 - p_{\mathbf{r}}^1\right) + \frac{i}{\omega L_{ty}}\left(p_{\mathbf{r+y}}^4 - p_{\mathbf{r}}^1\right) + i\omega C_0 p_{\mathbf{r}}^1 = 0, \quad (4)$$

in which p^j with $j = 1, 2, 3, 4$ represents the sound pressure at different cavities in the unit cell. Equations for other cavities can be expressed in the same way. Considering the Bloch boundary conditions, the equation takes the form of

$$(iwC + \frac{1}{iw}W)p = 0, \quad (5)$$

where

$$C = C_0 \mathbf{I}_{4\times4}, W = H - (\frac{2t + t_x + t_y}{L})\mathbf{I}_{4\times4}, L = L_t/t = L_x/t_x = L_y/t_y \quad (6)$$

and

$$H = \frac{1}{L}\begin{pmatrix} 0 & 0 & t_x + te^{ik_x} & t + t_y e^{ik_y} \\ 0 & 0 & t_y + te^{-ik_y} & t + t_x e^{-ik_x} \\ t_y + te^{ik_y} & t + t_x e^{ik_x} & 0 & 0 \\ t_x + te^{-ik_x} & t + t_y e^{-ik_y} & 0 & 0 \end{pmatrix}. \quad (7)$$

As a result, the band structures of the proposed acoustic model can be obtained by solving the eigenvalue problem

$$H\varphi = F(\omega)\varphi, F(\omega) = \left(\frac{2t + t_x + t_y}{L} - \omega^2 C_0\right), \quad (8)$$

in which $\varphi = [p^1, p^2, p^3, p^4]^T$ indicated the field distribution of acoustic pressure for each eigenfrequency. Clearly, Eq. 7 takes the identical form compared with the TBM in the main text Eq. (1).

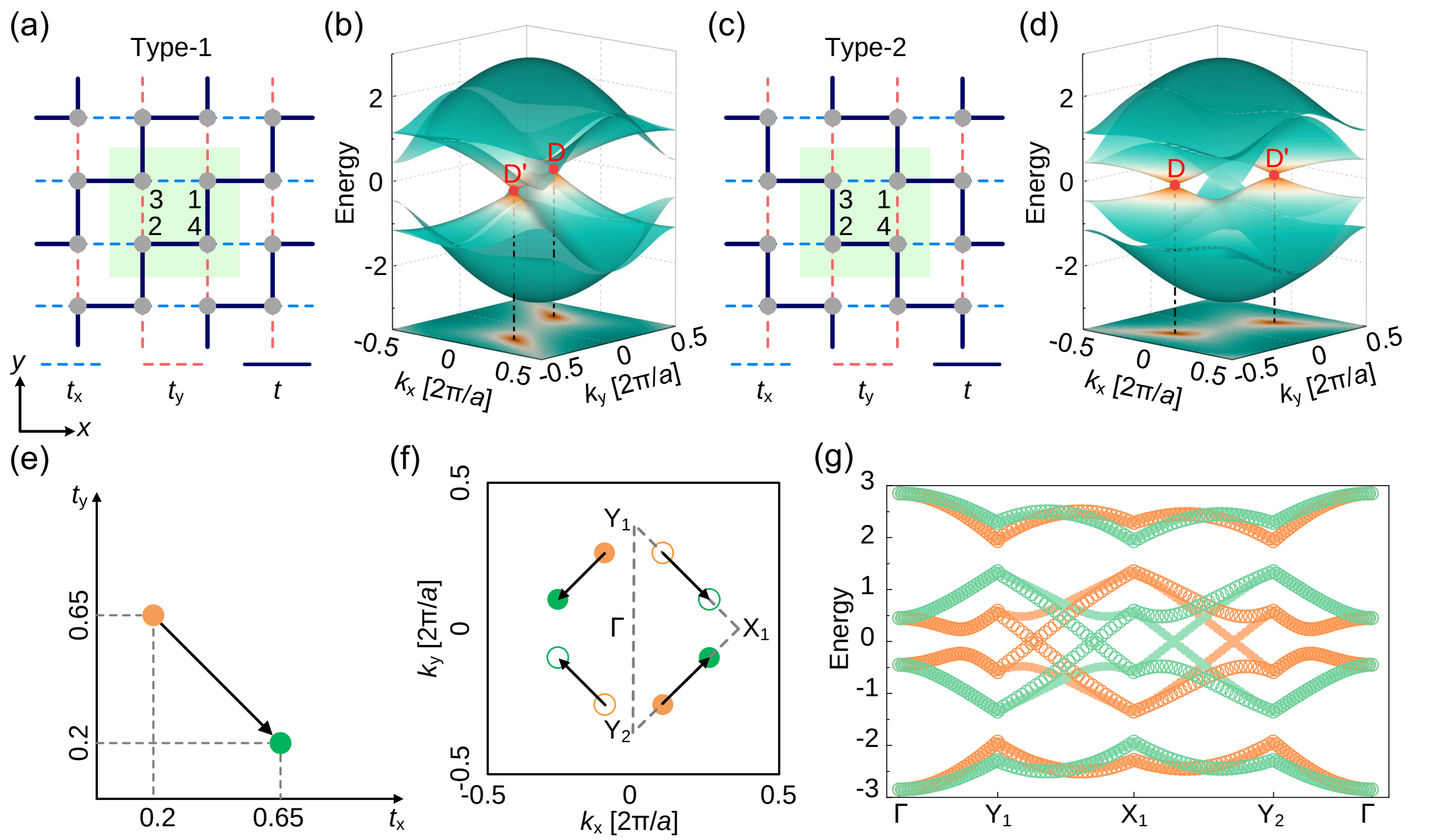

Figure S1. Tilted 2D SSH models supporting movable DCs. (a) Schematic of the type-1 2D SSH lattice. The unit cell consists of 4 sites. The solid lines denote couplings with constant hopping amplitudes of $t = 1$, while the blue (red) dashed lines denote couplings with varying hopping amplitudes of t_x (t_y) along the x (y) direction. (b) Band diagram calculated for the type-1 lattice with $t_x = 0.2$ and $t_y = 0.65$. (c,d) same as (a,b) but for the type-2 tilted SSH lattice. (e) Illustration of the path along which the coupling strength varies. (f) The positions of the DCs shift accordingly for the type-1 (solid dots) and type-2 (hollow circles) SCs with the parameter variations in (e). (g) Band diagrams calculated along the gray dashed path shown in (f) for four different scenarios.

Sect. 2. MOVED DCS ACROSS THE ENTIRE BZ

In this section, we demonstrate that the positions of DCs can be moved to any desired points within the first BZ in our proposed sonic crystals. Taking the type-1 lattice as an example, its effective Hamiltonian can be expressed as

$$H(\mathbf{k}) = \begin{pmatrix} 0 & q(\mathbf{k}) \\ q^{\dagger}(\mathbf{k}) & 0 \end{pmatrix}, \qquad \text{with} \quad q(\mathbf{k}) = \begin{pmatrix} t_x + te^{ik_x} & t + t_y e^{ik_y} \\ t_y + te^{-ik_y} & t + t_x e^{-ik_x} \end{pmatrix}. \tag{9}$$

The off-diagonal blocks in $H(\mathbf{k})$ indicate the chiral symmetry of $\Gamma H(\mathbf{k})\Gamma = -H(\mathbf{k})$ with $\Gamma = \tau_3 \otimes \sigma_0$, where τ and σ are Pauli matrices. Then the energy bands can be obtained from Eq. 9 as

$$E_{\eta}^{\pm}(\mathbf{k}) = \pm\sqrt{A_{\eta}^{2}(\mathbf{k}) + B_{\eta}^{2}(\mathbf{k})} = \pm\left|\varepsilon_{\eta}(\mathbf{k})\right| \tag{10}$$

where $A_{\eta}(\mathbf{k}) = (t+t_x)\cos\frac{k_x}{2} + \eta\,(t+t_y)\cos\frac{k_y}{2}$, $B_{\eta}(\mathbf{k}) = (t-t_x)\sin\frac{k_x}{2} - \eta\,(t-t_y)\sin\frac{k_y}{2}$, and $\varepsilon_{\eta}(\mathbf{k}) = A_{\eta}(\mathbf{k}) + iB_{\eta}(\mathbf{k})$ with $\eta = \pm 1$ for different bands. Due to the presence of chiral symmetry, the conduction and valence bands touch at zero energy. As a result, the existence of DCs yields the condition of $A_{\eta}(\mathbf{k}) = B_{\eta}(\mathbf{k}) = 0$. Solving these condition equations, we obtain $k_{x/y}$ which can be expressed as

$$k_{x/y} = 2\arccos\sqrt{\frac{\left(t+t_{y/x}\right)^{2}(2t - t_x - t_y)}{4t\left(t^{2} - t_x t_y\right)}}. \tag{11}$$

This equation holds when $|t_x + t_y| < 2t$ and $t_x \neq t_y$. A pair of DCs can be found at $\mathbf{k}_{\pm} = \pm(k_x, -k_y)$ if $t > t_{x/y}$. Otherwise, for $t < t_x$ or $t < t_y$, the pair of DCs appear at $\mathbf{k}_{\pm} = \pm(k_x, k_y)$. Based on Eq. 11, the coupling strengths can be modulated to alter the DC position. Note that the energy bands of the type-2 lattice could be derived in the same way as Eq. 10, with the only difference being $B_{\eta}(\mathbf{k}) = (t-t_x)\sin\frac{k_x}{2} + \eta\,(t-t_y)\sin\frac{k_y}{2}$. Consequently, the positions of the DCs are changed to $\mathbf{k}_{\pm} = \pm(k_x, k_y)$ with $t > t_{x/y}$, or to $\mathbf{k}_{\pm} = \pm(k_x, -k_y)$ with $t < t_x$ or $t < t_y$, where $k_{x/y}$ remains the same as Eq. 11. We demonstrate that titling describes the diagonally or off-diagonally distributed coupling patterns in SSH lattice, of which the degree can be quantified as $\sigma_x = t_x/t$ and $\sigma_y = t_y/t$. Consequently, the DC displacements in k space can be quantified as a function of tilting as

$$k_{x/y} = 2\arccos\sqrt{\frac{\left(1+\sigma_{y/x}\right)^{2}(2 - \sigma_x - \sigma_y)}{4\left(1 - \sigma_x\sigma_y\right)}} \tag{12}$$

for the type-1 and type-2 lattices according to Eq. 11. Setting the constant coupling strength as $t = 1$, DCs can be found in the BZ when $|t_x + t_y| < 2$, indicating the semimetal phase with DCs which is delineated by the region between the parallel lines $t_y = 2 - t_x$ and $t_y = -2 - t_x$ in Fig. S2a. Within this semimetal phase region, the DCs' locations evolve continuously with the hopping parameters. When $|t_x + t_y| > 2$, the model transitions into the insulator phase. Figure S2b illustrates the positions of the DCs while scanning the coupling strengths in the shaded region of Fig. S2a with $-3 \leq t_y \leq 3$. Additionally, when $t_x = t_y$, the band diagram transitions from containing DCs to forming a degenerate nodal line along $k_x = -k_y$. Notably, the type-1 lattice does not support DCs on the line $k_x = -k_y$. Employing a similar analytical method, the positions of the movable DCs in the type-2 lattice are visualized in Fig. S2c, showcasing the absence of DCs along the line $k_x = k_y$. However, by combining the characteristics of the type-1 and type-2 patterns, we can flexibly adjust the DCs' positions to any location

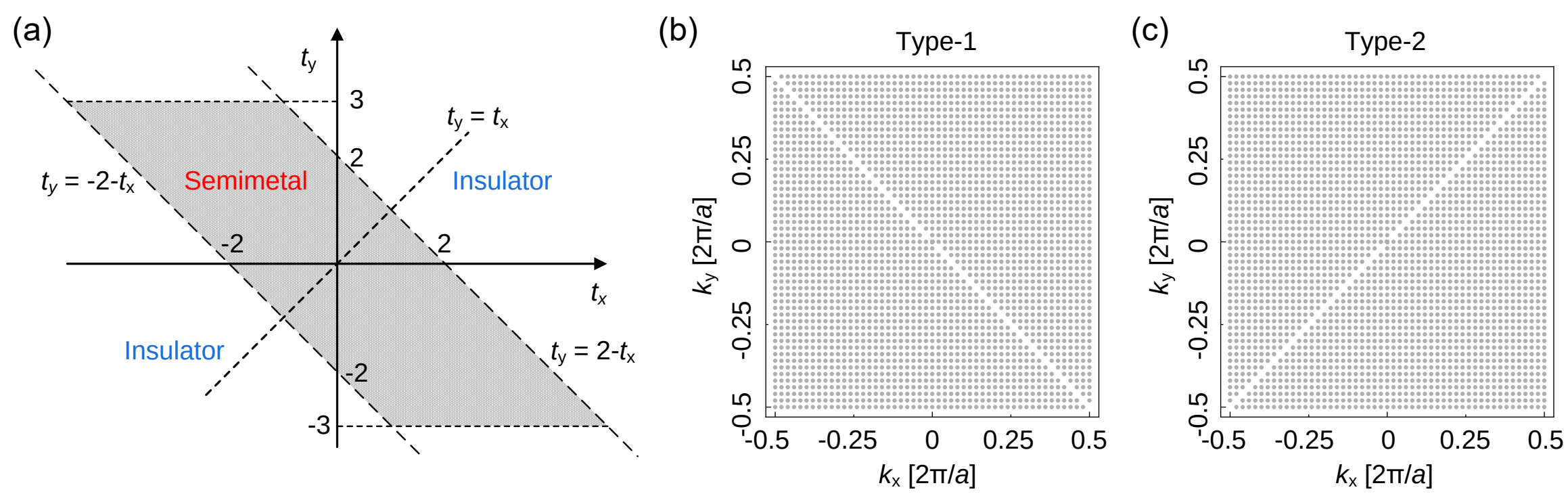

Figure S2. Moved DCs across the entire BZ. (a) Phase diagram of the tilted 2D SSH models in the (t_x, t_y) parameter space. The semimetal phase, accommodating a pair of DCs, is delineated between two dashed lines. The shaded region labels the parameter range selected for investigating the mobility of DCs. (b,c) Located positions of DCs for the (b) type-1 and (c) type-2 lattices, respectively, while scanning the parameters within the shaded region in (a).

Sect. 3. MATERIALS AND METHODS

A. Experimental Design

The SC was precision-fabricated using epoxy resin via 3D printing. The sample in main text Fig. 5a consisted of $6a \times 20a$ unit cells and was covered with a plexiglass plate on top. Two parallel semicircle plexiglass plates with the interval of 13.2 mm were used to form the free space, while sponges were placed along the exterior boundary to prevent reflections. Experimental setups utilized a loudspeaker (ENPILL PD-2121) with a 3D-printed pipe acting as a point sound source. Local pressure fields were captured by inserting a 1/4-inch condenser microphones (BSWA TECH Model: MPA-416) at specific positions on the top plate. Microphone outputs were acquired using a digitizer (NI PXI-4498) and processed by LabVIEW program. To demonstrate the far-field directional radiation of the topological acoustic antenna, the distance between the exit of the topological interface and the measuring microphones was chosen to be 0.95 m. Angular scans ranging from -89° to 89° were carried out with an increment of 2°.

B. Statistical Analysis

The numerical results presented in this work were obtained using the commercial finite-element software COMSOL Multiphysics. The mass density of the background air was set as 1.15 kg/m^3 with a sound speed of 351.87 m/s after considering the temperature of $35\,^\circ$C during experiments. Due to the large acoustic impedance mismatch between air and the 3D printed epoxy resin materials, the walls of the cavities and tubes were modeled as acoustic hard walls. For band structure calculations, Floquet periodic conditions were applied on the unit cells or strip boundaries. While calculating the radiated far-field patterns of pressure fields, cylindrical wave radiation and far-field calculation conditions were imposed.

Sect. 4. REAL-TIME MODULATION OF DCS' POSITIONS

In this section, we present a feasible experimental setup to achieve real-time modulation on the positions of DCs. As demonstrated in the main text, DCs in the Brillouin zone can be moved via changing the real-space pattern and strength of the couplings. To this end, we design a tube-cavity system based on the first-order acoustic resonance (see Fig. S3a), where the coupling strength can be conveniently modified by varying the vertical positions of the tubes connecting the adjacent sound cavities. Each tube has a width of $w = 2$ cm and a height of $h_{\mathrm{t}} = 0.3$ cm, while the cavities possess a side length of $b = 3.5$ cm and a height of $H = 6$ cm, with a separation distance of $d = 6.2$ cm between them. The first-order resonance mode of the cuboid functions as the on-site orbital with a frequency of f_0. This dual-resonator system constitutes the fundamental unit of the proposed SC, for which the Hamiltonian can be given by

$$H = \begin{pmatrix} f_0 & t \\ t & f_0 \end{pmatrix}. \tag{13}$$

The eigenvalues of the Hamiltonian are $f_1 = f_0 - t$ and $f_2 = f_0 + t$, and the corresponding pressure field distributions of the symmetric/anti-symmetric eigenstates are shown in the right panel of Fig. S3a at $h_{\mathrm{c}} = h_{\mathrm{t}}/2$. Subsequently, by adjusting the position of the coupling tube h_{c}, the variations in eigenfrequencies are observed in Fig. S3b. Through characterization of the coupling strength as $t = (f_2 - f_1)/2$, Fig. S3c confirms the effective modulation of coupling strength by manipulating the vertical placement of the coupling tube. The maximum coupling strength is attained when the tube is positioned at the topmost part of the cavity, whereas nearly zero strength is achieved when the tube is centralized within the cavity.

Based on the methodology discussed above, we have designed a tilted 2D SSH SC, as shown in Fig. S3d, to achieve real-time modulation on the DCs' positions. The y-direction couplings are classified into two sets as labeled by the orange and pink colors, of which the locations can be modulated through h_{cy1} and h_{cy2}, respectively. Similarly, the x-direction couplings are divided into two sets labeled by blue and cyan colors, with their positions controlled by h_{c} and h_{cx}, respectively. As illustrated in Fig. S3e, these fours sets of coupling tubes can be independently vertically adjusted using movable poles along grooved tracks [1]. Consequently, by maintaining $h_{\mathrm{c}} = h_{\mathrm{cy2}}$ and adjusting the locations of other coupling tubes h_{cx} and h_{cy1}, the type-1 tilted 2D SSH model can be implemented. Conversely, retaining $h_{\mathrm{c}} = h_{\mathrm{cy1}}$ while modifying the locations of other coupling tubes h_{cx} and h_{cy2} leads to the realization of the type-2 tilted 2D SSH model. For instance, setting

the parameters as $h_\mathrm{c} = h_\mathrm{cy2} = h_\mathrm{t}/2$ represents the type-1 model, with $h_\mathrm{cx} = h_\mathrm{t}/2$ and varied h_cy1. The band diagrams with $k_y = 0$ are depicted in Fig. S3f, where gray and blue dots correspond to the outcomes with $h_\mathrm{cy1} = 0.1$ cm and 0.3 cm, respectively, showcasing the movement of DCs within the BZ. This approach for adjusting the positions of DCs enables the realization of versatile real-time topological functional devices.

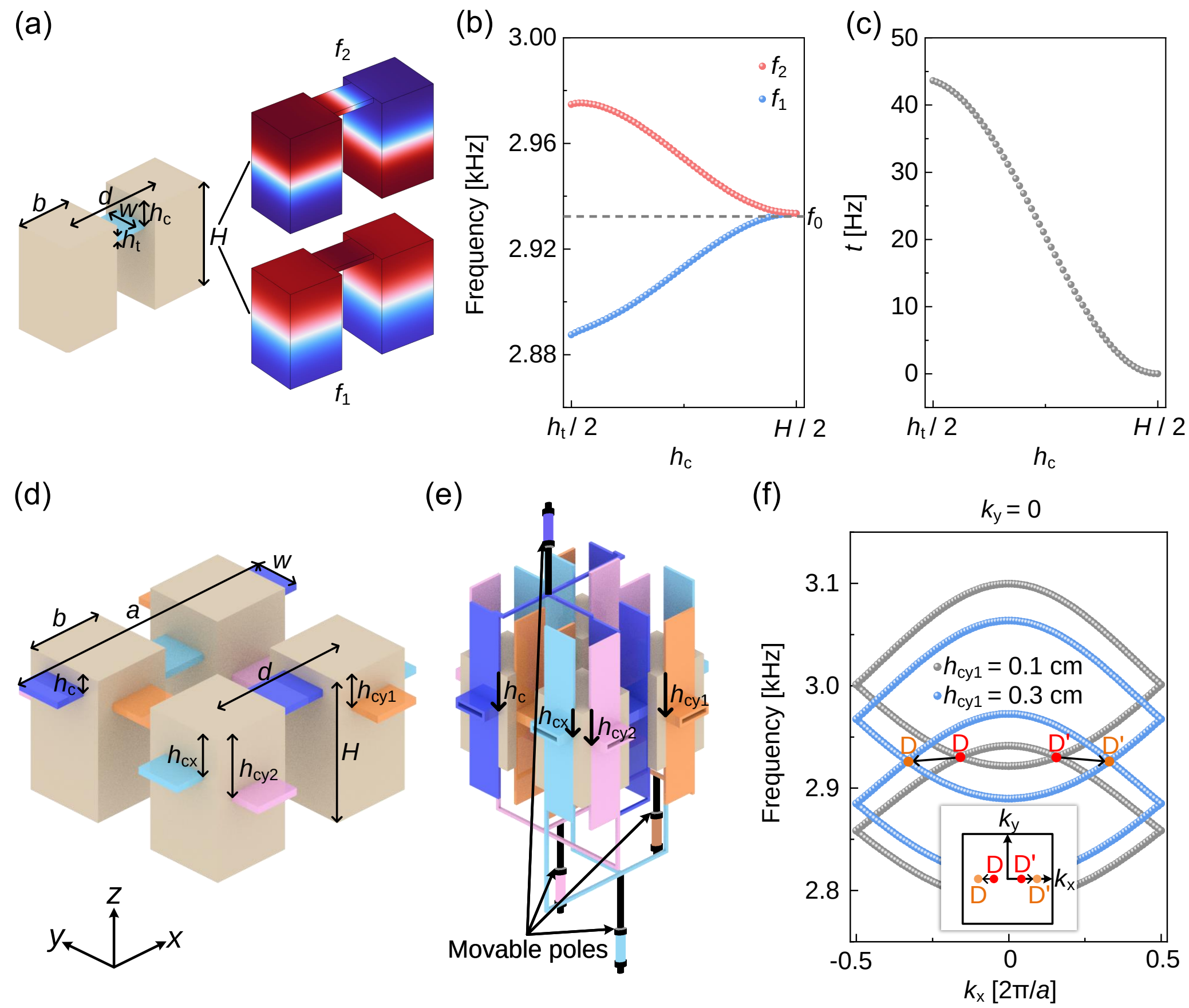

Figure S3. Real-time modulation of the DCs' positions. (a) Schematic of two tube-coupled resonant cavities. Right panels show the split modes. (b,c) Variations of eigenfrequencies for different modes (b) and of coupling strength (c) against the position of the movable coupling tube. (d) Schematic of the unit cell for the tilted 2D SSH SC. (e) Experimental setup to achieve the real-time modulation of the coupling strength via movable poles. (f) Band diagrams for the SCs while varying h_cy1.

Sect. 5. DISPERSION RELATIONS OF RIBBON-SHAPED 2D SCS

Figures S4a and S4b depict the calculated dispersion relations of the topological edge states (red dots) for the structures in the main text Fig. 4c and 4f, respectively. It is proven that the edge state propagating from left to right are projected by the valley D.

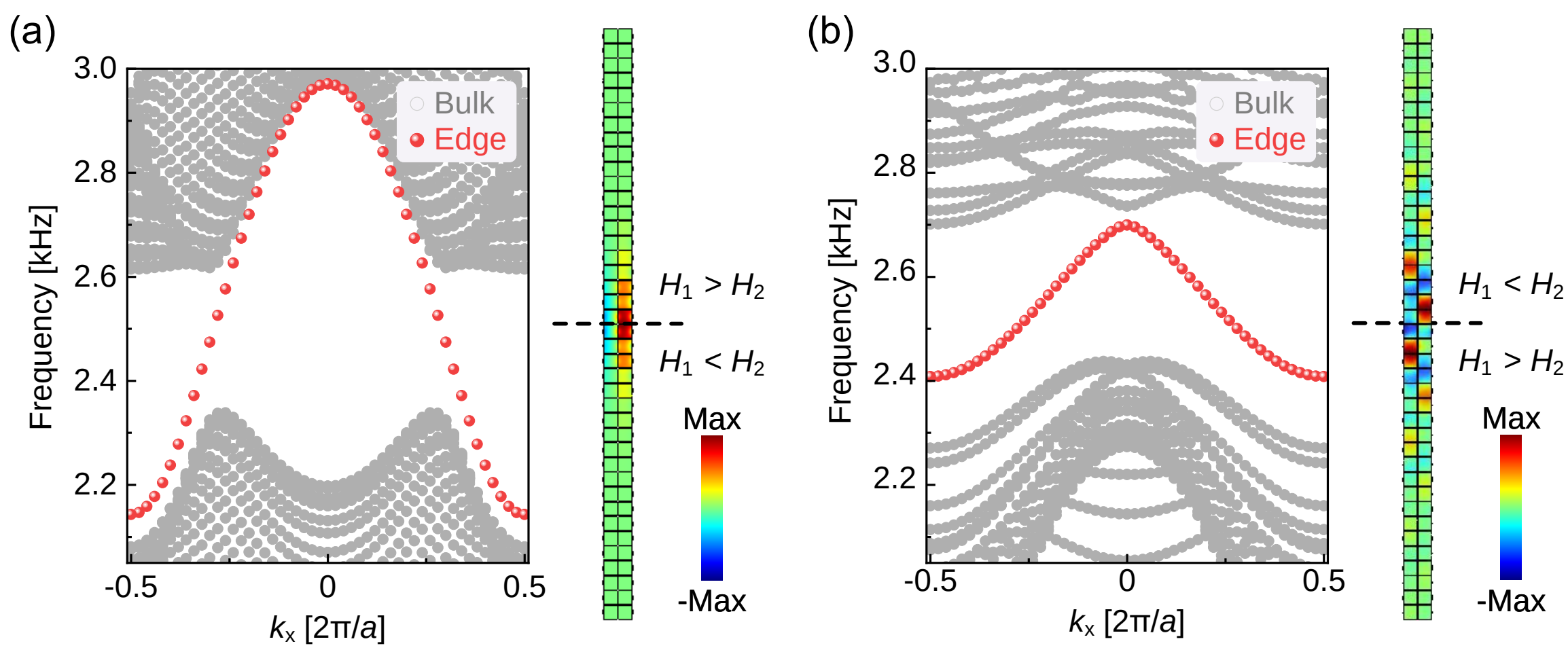

Figure S4. Dispersion relations of the ribbon-shaped 2D SCs. (a) Simulated dispersion relation of the ribbon-shaped SCs along the k_x direction for the type-2 SC with $w_x = w$ and $w_y = 0.12w$. (b) Same as (a) but for type-1 SC with $w_x = 0.16w$ and $w_y = 1.79w$.

Sect. 6. ANALYSIS ON THE INFLUENCE OF INHERENT LOSS

To investigate the effect of loss, we introduce an imaginary part to the sound speed as $c = 351.87 \times (1 + 0.01i)$ m/s in our simulations to act as inherent viscous loss in realistic environment. Figures S5(a)–S5(c) present the results for the topological omnidirectional antenna, showing that the simulations incorporating losses yield better agreement with the experimental data. In contrast, the simulated results of the sound Klein tunneling effect do not appear to be significantly affected by the introduction of losses, as the two corresponding curves in Figs. S5(d) and S5(e) almost coincide. As a result, we have proven that the viscous loss in the system does not affect the DC stability and the functionality of the proposed topological devices, such as the all-angle topological omnidirectional antenna and Klein tunneling effects.

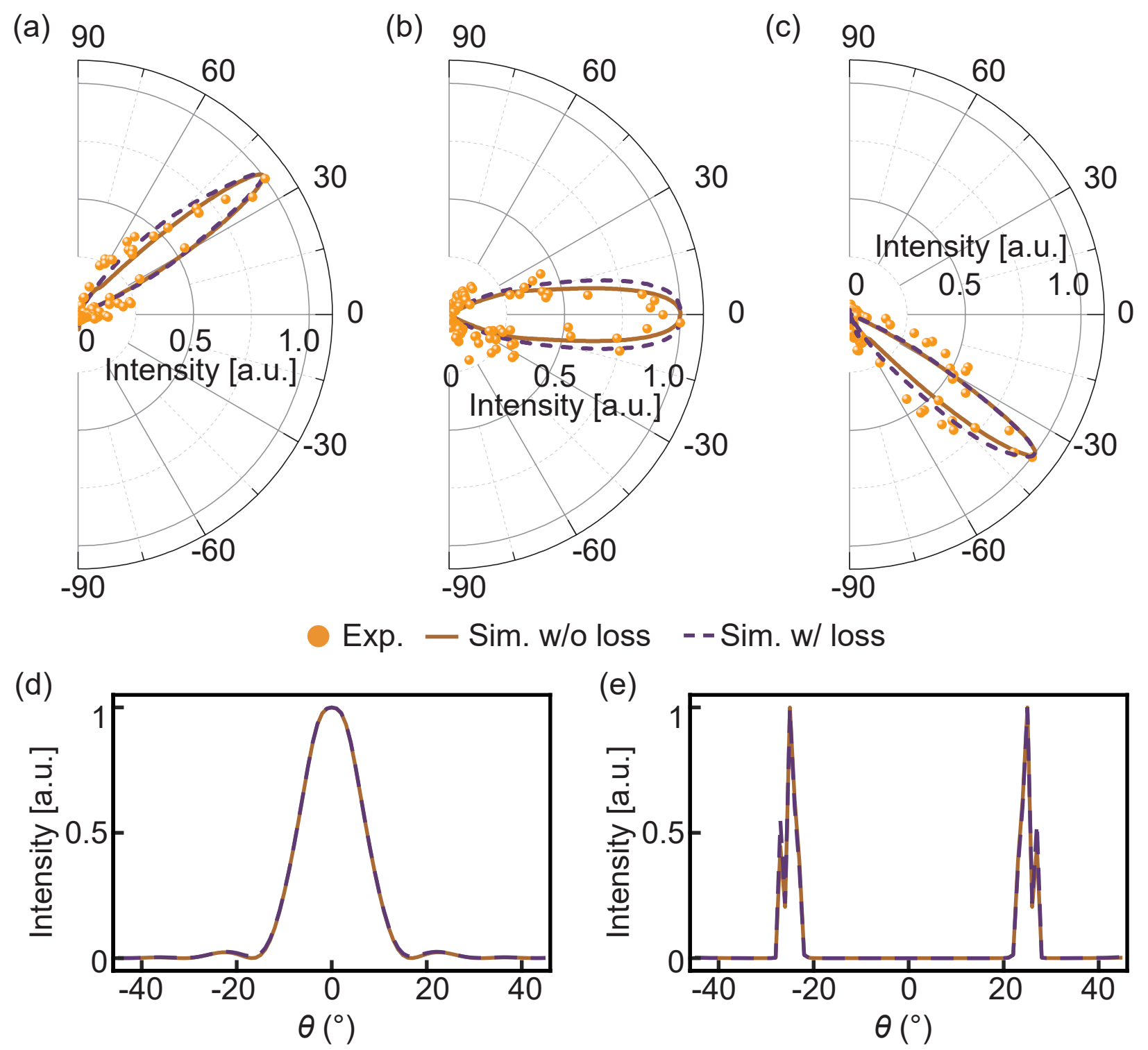

Figure S5. Influence of loss. (a)-(c) Simulated far-field radiation patterns without (brown solid curves) and with loss (purple dashed curves), together with the experimentally measure data (dots) for three kinds of structures. (d) and (e) Same as (a)-(c) but for the analysis on the Klein tunneling effect.

Sect. 7. DIRECTIONAL RADIATION TOWARDS LARGER ANGLES

It is crucial to reiterate that the manipulation of DCs over the entire BZ allows for the adjustment of radiation angles in full space. Thus, using the proposed methodology of topological antenna, we could obtain arbitrary directional angles. In the main text, three scenarios are presented, reporting radiation angles of $-36.8°$, $0°$, and $36.8°$, both numerically and experimentally. Herein, we demonstrate the capability of our movable DCs to generate large-angle directional sound radiations. Figures S6a and S6b illustrate directional sound beams at angles of $\pm 61.8°$ based on the type-1 structures with dimensions $h = 6$ mm, $b = 32$ mm, $w = 12$ mm, $w_x = 0.3w$, and $w_y = 0.87w$. By tuning the coupling strengths to $w_x = 0.12w$ and $w_y = 1.35w$, the directional emissions into free space of out-coupled edge states are shown in Figs. S6c and S6d, where the radiation angles extending up to $\theta = \pm 90°$.

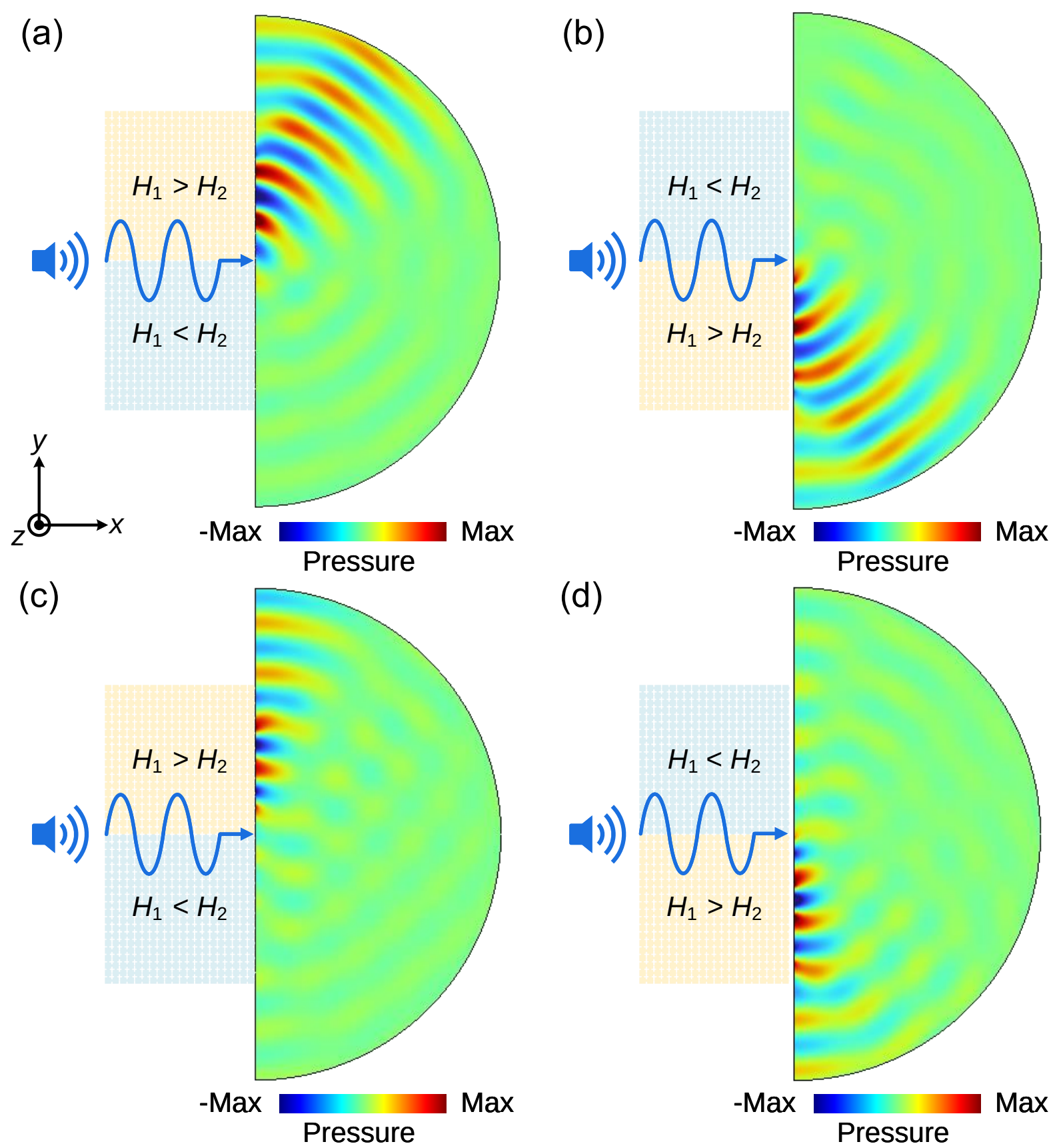

Figure S6. Directional acoustic antenna with large radiation angles. Simulated distributions of pressure fields for realizing the directional radiation with the angle of (a) $61.8°$, (b) $-61.8°$, (c) $90°$, and (d) $-90°$ at the frequency of $f = 1.45$ kHz.

Moreover, we have provided other four directionally radiative far-field patterns towards large angles as illustrated in Fig. S7. Figure S7(a) and S7(b) show the directional sound beams with radiation angles of $\pm 32.1°$ based on the type-1 structures with geometric parameters of $h = 6$mm, $b = 32$ mm, $w = 12$ mm, $w_x = 0.55w$ and $w_y = 0.9w$. By tuning the coupling strengths to $w_x = 0.1w$ and $w_y = 1.5w$, the directionally radiated acoustic pattern of the out-coupled edge states are described in Figs. S7(c) and S7(d) with the radiation angles up to $\pm 70.6°$.

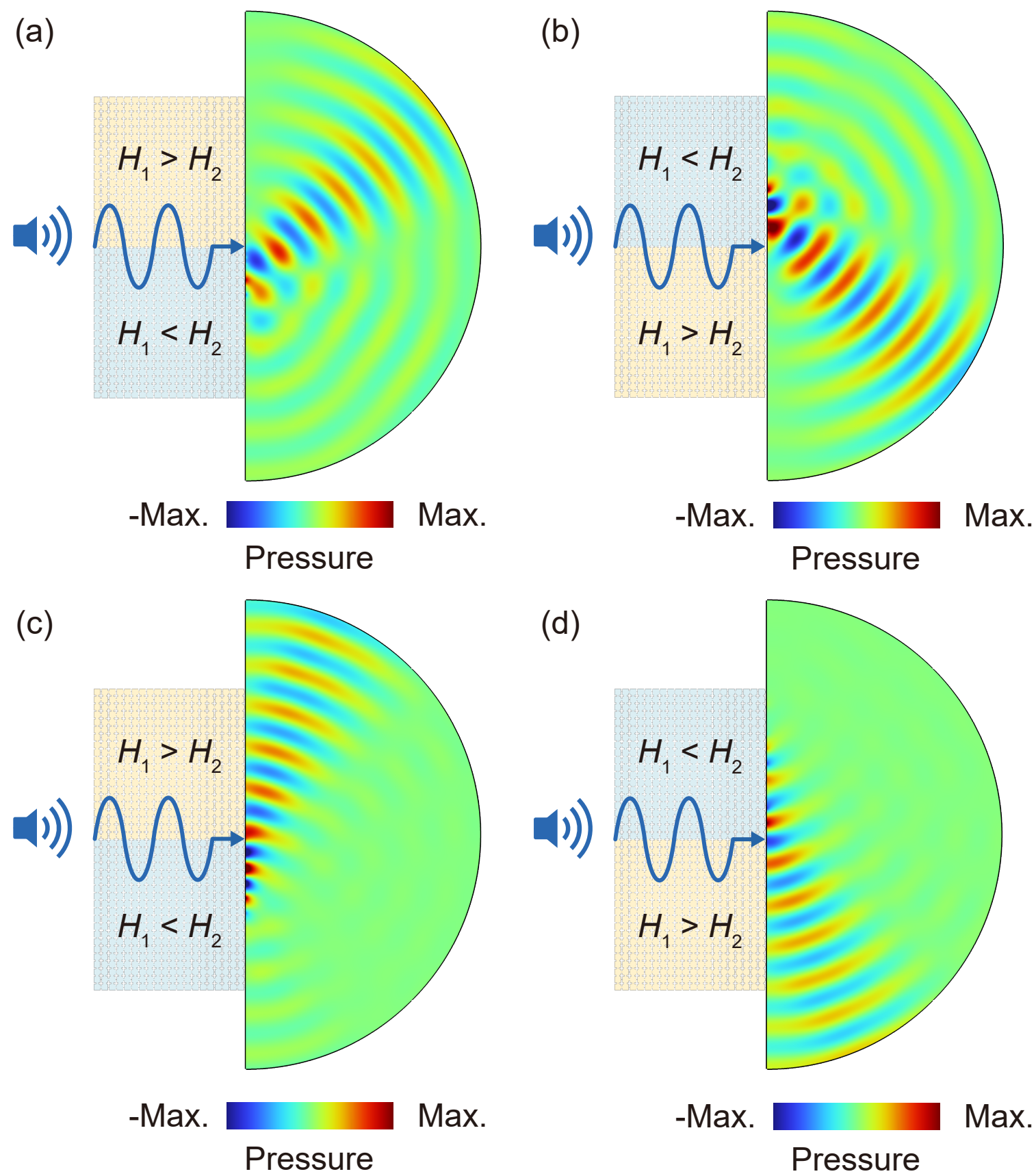

Figure S7. Simulated distributions of pressure fields for realizing the directional radiation with the angle of (a) $32.1°$, (b) $-32.1°$ at the frequency of 1.5 kHz and (c) $70.6°$, (d) $-70.6°$ at the frequency of 1.7 kHz.

Sect. 8. ROBUSTNESS ANALYSIS

Here, the defects are introduced by inserting acoustically rigid cubic blocks with the size of $d =$ 8 mm and $h_0 = 3.5$ mm into the randomly distributed cavities. First, we focus on the robustness of the topological antenna against defects. As illustrated in the left panel of Fig. S8(a), the disordered cavities are located only near the domain wall. The right panel indicates that more defects are introduced into the bulk of structure. Figure S8(b) shows the simulated far-field radiation patterns without defects and with the above two kind of defects for the topological antennas sharing the identical geometric parameters as the ones in the main text Fig. 5(b). One can clearly observe that the curves corresponding to the perturbed systems coincide with the results without disordered defects perfectly, indicating strong robustness. Leveraging the similar analysis, the robustness of the Klein tunneling effect is also explored via simulations as shown in Figs. S8(c) and S8(d), which supports $0°$ Klein tunneling with the identical structural details with the one in the main text Fig. 6(d). It further verifies that our propose systems still achieve the desired effects despite the introduced onsite defects.

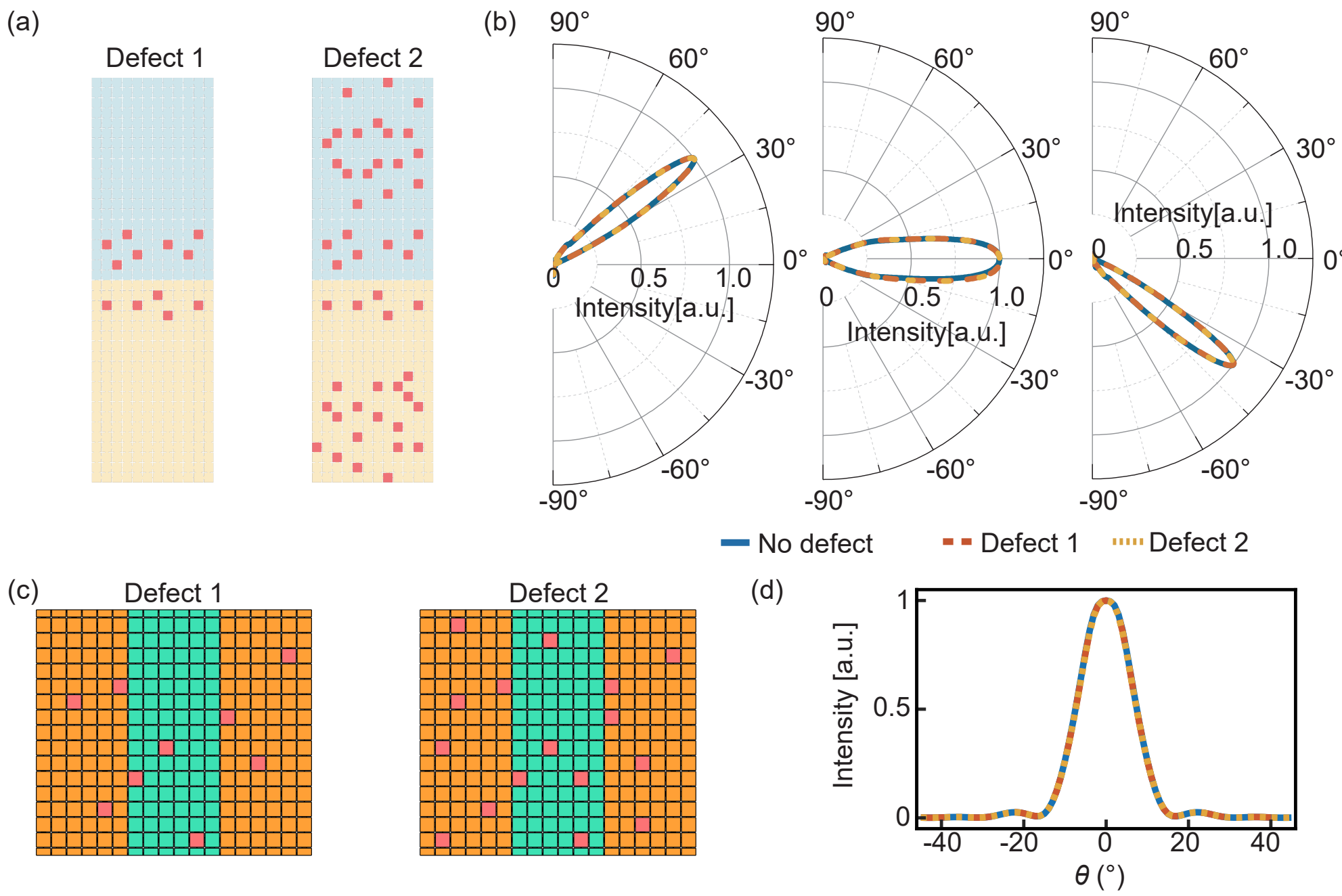

Figure S8. Robustness against onsite defects. (a) Schematics of the perturbed topological antenna structures with different numbers of disordered cavities. (b) The simulated acoustic far-field radiation patterns without defects, with defect 1 and with defect 2 as labeled in (a), which are marked by the blue solid, red dashed and yellow dotted curves, respectively. (c) and (d), Same as (a) and (b) but for the robustness analysis of acoustic Klein tunneling effect. Red colored cavities in (a) and (c) label the disordered cavities.

Sect. 9. THE TRANSMISSION COEFFICIENTS VS. INCIDENT ANGLES FOR THE SOUND KLEIN TUNNELING EFFECT

By setting the geometric parameters of SC as $w_x = w = 12$ mm and $w_y = 0.09w$, the paired DCs in regions A and B are located at D $(-0.3, 0)\frac{2\pi}{a}$ and D′ $(0.3, 0)\frac{2\pi}{a}$. While the incident angle of sound wave is gradually increased, the averaged output intensities (I_{out}) are extracted from the red dashed line as shown in Fig. S9(a). The results extracted from this line without acoustic heterostructure are regarded as the input intensities (I_{in}). Then the transmission coefficient vs. angle can be achieved as $I_{\text{out}}/I_{\text{in}}$ as depicted in Fig. S9(b), indicating 0° Klein tunneling effect. Figure S9(c) further demonstrates the simulated transmission coefficient for the structures with $w_x = 0.24w$ and $w_y = 0.85w$ supporting the DCs located at D $(-0.04, 0.26)\frac{2\pi}{a}$ and D′ $(0.04, -0.26)\frac{2\pi}{a}$, which shows 25° Klein tunneling effect.

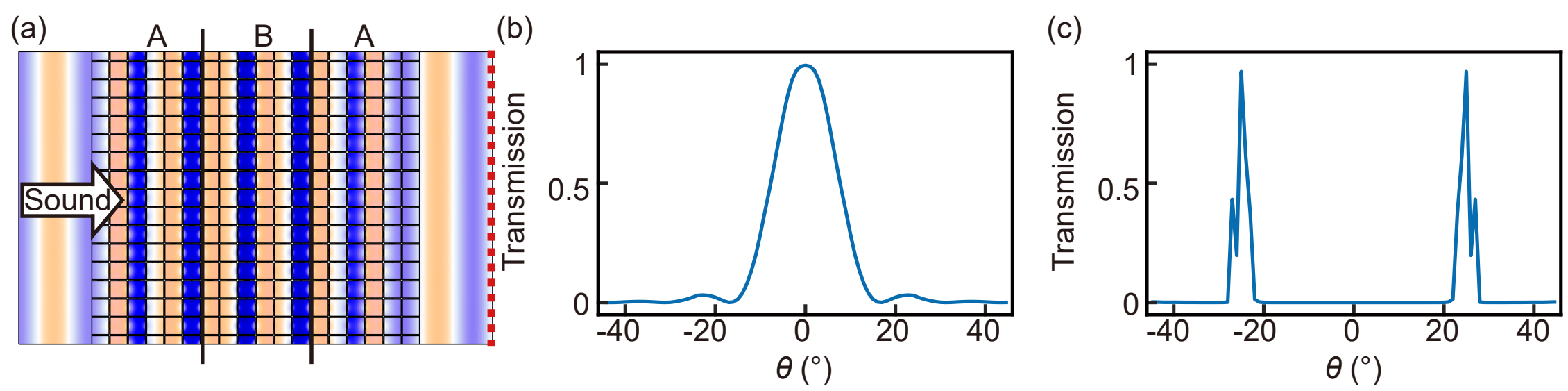

Figure S9. Transmission coefficient vs. incident angle. (a) Illustration of the acoustic heterostructure for realizing Klein tunneling effect. Calculated transmission coefficient vs. angle for the structures supporting DCs at (b) D $(-0.3, 0)\frac{2\pi}{a}$/D′ $(0.3, 0)\frac{2\pi}{a}$ and (c) D $(-0.04, 0.26)\frac{2\pi}{a}$/D′ $(0.04, -0.26)\frac{2\pi}{a}$, respectively.

Sect. 10. AN EXAMPLE OF WAVE VECTOR-TUNABLE VALLEY FILTER

Taking the valley filters as an example, we conduct additional simulations to verify its wavevector-tunable characteristics in Fig. S10. As shown in Fig. S10(a), a topological interface formed by two type-2 SCs with different cavity heights is placed between two gapless SCs A with uniform cavity height of $H_1 = H_2$. Coupling parameters are set as $w_x = 0.16w$, and $w_y = 1.79w$, which is same as the main text Fig. 3(a). Figure S10(b) illustrates the simulated acoustic pressure field, which is excited by a point source placed at the left termination of part A with the frequency of 2.6 kHz. The derived Fourier spectra in the first BZ for three regions are depicted in Figs. S10(c)-S10(e). It can be observed that several wave vectors are excited within the left incident part. However, only the wave vectors matching with the specific Dirac point (D) can propagate through the middle part and the sound waves with DC-dependent wave vectors can be filtered. We also provide the simulations for the acoustic valley filter based on the type-1 structure in Figs. S10(f)-S10(j), which share the identical coupling parameters with the case in the main text Fig. 4(b), demonstrating the capability for our structure to filter sound waves with the wave vectors corresponding to other translated DCs.

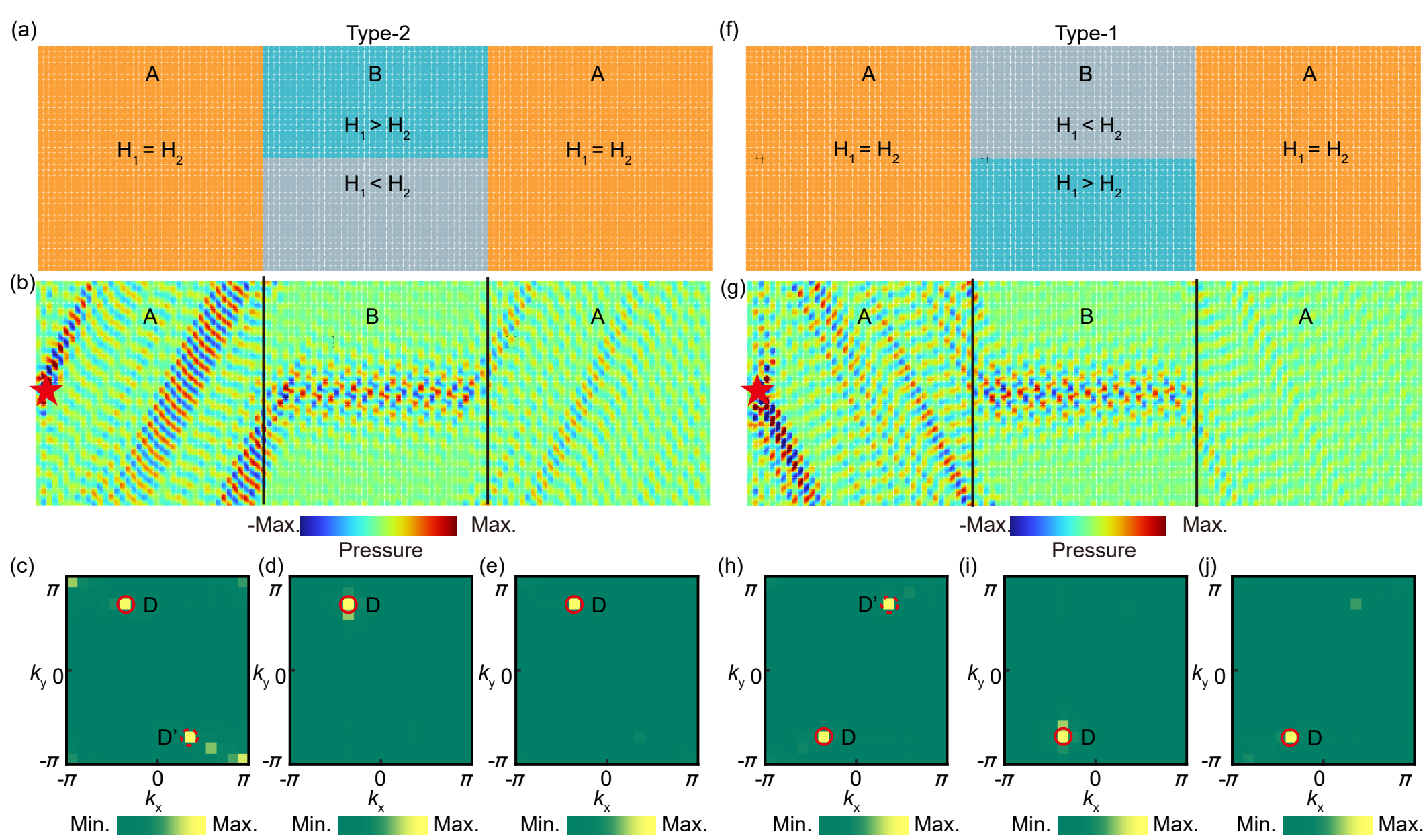

Figure S10. Tunable valley filter. (a) Schematic of the valley filter based on type-2 SC. (b) Simulated pressure fields at the frequency of 2.6 kHz. (c)-(e) The Fourier spectra corresponding to three regions. The DCs are marked by red circles. (f)-(j) Similar to (a)-(e) but for type-1 SC with the translated DCs.

[1] J.-P. Xia, D. Jia, H.-X. Sun, S.-Q. Yuan, Y. Ge, Q.-R. Si, and X.-J. Liu, Programmable coding acoustic topological insulator, Adv. Mater. **30**, 1805002 (2018).
